# Insulin/IGF1-PI3K-dependent nucleolar localization of a glycolytic enzyme – phosphoglycerate mutase 2, is necessary for proper structure of nucleolus and RNA synthesis

**DOI:** 10.18632/oncotarget.4044

**Published:** 2015-05-08

**Authors:** Agnieszka Gizak, Marcin Grenda, Piotr Mamczur, Janusz Wisniewski, Filip Sucharski, Jerzy Silberring, James A. McCubrey, Jacek R. Wisniewski, Dariusz Rakus

**Affiliations:** ^1^ Department of Animal Molecular Physiology, Wroclaw University, Cybulskiego, Wroclaw, Poland; ^2^ Department of Biochemistry and Neurobiology, Faculty of Materials Science and Ceramics, AGH University of Science and Technology, al. Mickiewicza, Kraków, Poland; ^3^ Department of Microbiology and Immunology, Brody School of Medicine at East Carolina University Greenville, NC, USA; ^4^ Biochemical Proteomics Group, Department of Proteomics and Signal Transduction, Max-Planck-Institute of Biochemistry, Am Klopferspitz, Martinsried, Germany

**Keywords:** squamous cell carcinoma, PGAM2, rRNA, ribosome assembly, multifunctional enzyme

## Abstract

Phosphoglycerate mutase (PGAM), a conserved, glycolytic enzyme has been found in nucleoli of cancer cells. Here, we present evidence that accumulation of PGAM in the nucleolus is a universal phenomenon concerning not only neoplastically transformed but also non-malignant cells. Nucleolar localization of the enzyme is dependent on the presence of the PGAM2 (muscle) subunit and is regulated by insulin/IGF-1–PI3K signaling pathway as well as drugs influencing ribosomal biogenesis. We document that PGAM interacts with several 40S and 60S ribosomal proteins and that silencing of PGAM2 expression results in disturbance of nucleolar structure, inhibition of RNA synthesis and decrease of the mitotic index of squamous cell carcinoma cells. We conclude that presence of PGAM in the nucleolus is a prerequisite for synthesis and initial assembly of new pre-ribosome subunits.

## INTRODUCTION

Over the last twenty years, it has been demonstrated that evolutionarily old enzymes of carbohydrate metabolism are present not only in cytoplasm but also in the cell nucleus or mitochondria. In these compartments, they are engaged in regulation of many basic cellular processes such as proliferation, differentiation or programmed cell death, and their biological activity is not necessarily restricted to catalytic activity.

One of them is phosphoglycerate mutase (PGAM, EC 5.4.2.1), a conserved, glycolytic enzyme which catalyses reversible conversion of 3-phosphoglycerate to 2-phosphoglycerate. Mammalian PGAM consists of two subunits, called muscle (M or PGAM2) and brain (B or PGAM1). PGAM has three isozymes: muscle-type (PGAM-MM), brain-type (PGAM-BB) homodimers or a heterodimer (PGAM-MB). In different tissues, various proportions of these dimers are detected, but most mouse cells contain the heterodimeric form [1].

In cancer cells, the enzyme appears to be a central element of a glycolytic macromolecular complex composed of enzymes of triose phosphate metabolism and regulated by lactate. Surprisingly, in these cells, PGAM was also found in nucleolar structures [2].

The nucleolus is traditionally associated with the synthesis and assembly of ribosome subunits. However, evidence has accumulated that this structure has additional regulatory functions. In human nucleolar proteome, one can find proteins involved not only in ribosome biogenesis but also in regulation of cell cycle and stress responses (reviewed in [3]). Proteomic studies have also demonstrated the presence of glycolytic and pentose phosphate pathway enzymes in nucleoli [4], but these authors did not discuss the physiological relevance of such localization. Instead, the authors' comments suggested that they expected a “classical” metabolic role of these enzymes in nucleoli.

To address the question concerning the role of PGAM in nucleolus, we have sought to identify signaling pathways regulating PGAM localization in this compartment and to identify nucleolar proteins interacting with the enzyme. We have also tested the effect of PGAM silencing on the nucleolar structure and function. We have observed that PGAM2 subunit is crucial for nucleolar localization of PGAM homo- and heterodimers. Results presented here demonstrate that PGAM is directed to nucleoli of neoplastically transformed and non-malignant cells by insulin/IGF-1/PI3K signaling pathway and appears to interact with ribosomal proteins. The silencing of the PGAM2 expression results in disturbance in nucleolar structure as well as in inhibition of RNA synthesis. Together, our findings suggest that PGAM2 is a crucial part of the mechanism of nucleolar response to growth factors and may be a target in some transformed cells which grow in response to aberrant IGF-1/PI3K signaling.

## RESULTS

### Detection of PGAM in nucleoli

**Figure 1:**
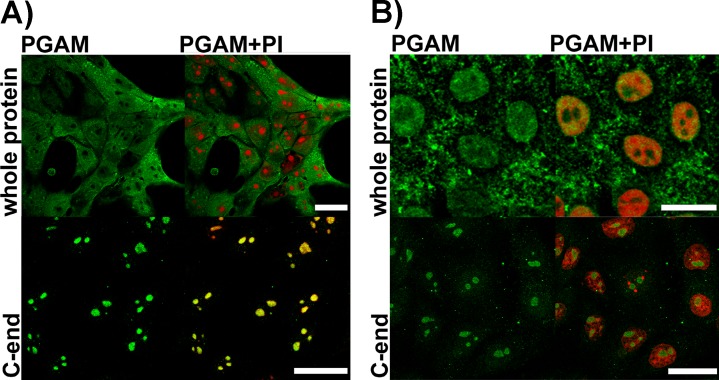
Detection of PGAM in KLN-205 cells with antibodies directed to whole PGAM protein or to C-terminal peptide of PGAM **A.** control conditions (scan parameters in red channel were set to emphasize nucleolar staining with propidium iodide – PI) **B**. RNase-treated cells. Bar=15 μm.

As we have demonstrated before [2], incubation of KLN-205 cells with polyclonal antibodies directed to the whole PGAM protein resulted in strong staining of cytoplasm, much weaker staining of nucleus and no staining of some subnuclear compartments (Figure 1A) of these cells. However, after incubation of the KLN-205 cells with antibody directed towards the C-terminal peptide of PGAM, the immunoreaction was found mainly in the subnuclear compartments (Figure 1A). These compartments were identified as the nucleoli since they were propidium-iodide(PI)-positive in untreated cells, but PI-negative in cells treated with RNase (Figure 1B).

**Figure 2:**
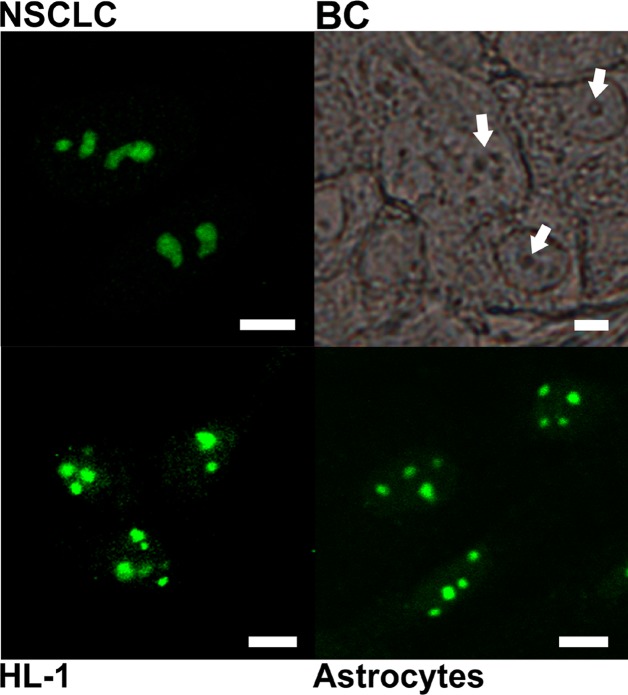
Subcellular localization of PGAM with the use of antibody directed to C-terminal peptide of the protein The localization was examined in cultures of non-small cell lung carcinoma (NSCLC) HL-1 cardiomyocytes, astrocytes and in breast cancer tissue section (BC). Arrows point nucleoli. Bar=5 μm.

Similar strong nucleolar staining was observed also in histological sections of human breast cancer (BC), in primary cultures of neoplastically transformed cells of human non-small cell lung carcinoma (NSCLC), and also in non-malignant cells (cultured mouse astrocytes and HL-1 cardiomyocytes), incubated with antibody against the C-terminal PGAM peptide (Figure 2).

It has been suggested that the observed differences in immunostaining between these two types of antibodies result from the inability of the antibodies against the whole PGAM molecule to detect its C-terminal peptide rather than from an unspecific reaction of the antibody against the C-terminus of PGAM with some nucleolar antigens [2]. However, this might imply that PGAM in nucleoli is tightly surrounded by other proteins or nucleic acids and in consequence, only the C-terminal region of the enzyme is available to antibodies.

To verify this, the KLN-205 cells were treated with RNase A prior to fixation and incubation with antibodies against the whole PGAM molecule. After such treatment nucleolar presence of PGAM was detected in about 60% of the cells (Figure 1B). However, at the same time, staining of the nucleoli with the PGAM C-terminus-specific antibodies became less intensive (Figure 1B). Together, this might indicate that although the nucleolar RNA is a factor reducing the availability of PGAM to antibodies, and it is also – a direct or indirect – binding partner of the protein.

To unequivocally confirm nucleolar localization of PGAM, we sought for the presence of the enzyme in sucrose gradient-isolated nucleoli of the KLN-205 cells using mass spectrometry. The analysis revealed the presence of peptides specific for the brain and muscle PGAM subunits (PGAM1 and 2), as well as PGAM5 (Supplementary Figure 1). PGAM5 lacks phosphoglycerate mutase activity acting instead as a Ser/Thr protein phosphatase [5] and was classified as a nucleolar polypeptyde folding factor [6].

### Identification of the signaling pathways regulating nucleolar localization of PGAM

**Figure 3:**
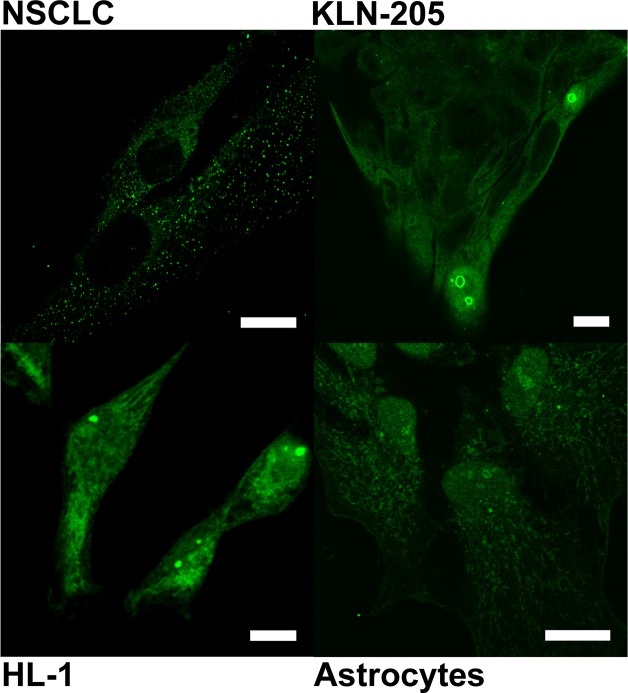
Localization of PGAM with the use of antibody against the C-terminal peptide in neoplastically transformed (NSCLC, KLN-205) and non-malignant (HL-1 cardiomiocytes, astrocytes) cells cultured in serum-free media Bar=10 μm.

In KLN-205 and NSCLC cells cultured in the absence of serum, immunocytochemical methods failed to detect PGAM in nucleoli of the majority of the cells (Figure 3), suggesting that regulation of the PGAM presence within these substructures was due to some serum-derived factors. In such conditions, antibodies directed to the C-terminus of PGAM stained mainly cytoplasm and, in about 1% of cells, outer region of nucleoli and also nucleus. In other types of examined cells, serum starvation also resulted in reduction of nucleolar PGAM and detection of the C-terminus of the protein both in cytoplasm as well as whole nucleus (Figure 3).

Not surprisingly, in the serum-deprived cultures, mitotic figures were not observed indicating cessation of proliferation (data not shown).

In line with this, the mass spectrometry analysis of nucleoli isolated from KLN-205 cells cultured in the absence of serum identified only the PGAM5-derived peptides (Supplementary Figure 1). Together, these results provided evidence that in serum-free culture conditions, PGAM was absent from nucleoli.

**Figure 4:**
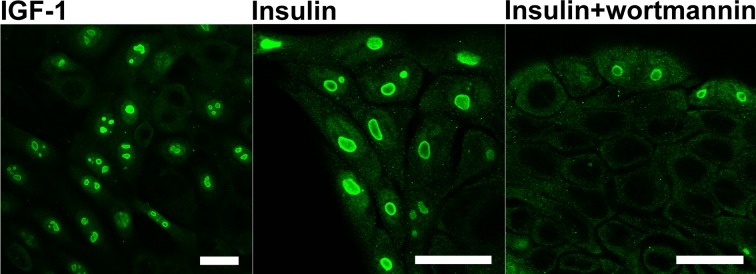
Localization of PGAM in KLN-205 cells in different culture conditions PGAM was localized with the use of antibody against the C-terminal peptide of the protein in the cells cultured in serum-free medium re-supplemented with IGF-1, insulin or insulin and inhibitor of PI3K (wortmannin). Bar=15 μm.

After 24h culture of the KLN-205 cells in the serum-free medium supplemented with insulin, the proliferation was resumed and PGAM re-appeared in nucleoli, as demonstrated with the use of antibodies directed to the C-terminal region of the enzyme. This re-appearance was relatively slow and in some cells, whole PGAM-positive nuclei were observed or PGAM was present mainly in outer region of nucleoli (forming cap-like structures) (Figure 4). Similar effects were obtained after supplementation of the serum-free medium with IGF-1 (Figure 4). However, if insulin was added together with an inhibitor of PI3K (wortmannin) which is a downstream target of insulin signaling, nucleolar localization of PGAM was not detected (Figure 4). In majority of the cells, the serum withdrawal effected also changes of nucleolar amount and morphology: instead of 2-3 nucleoli, only one enlarged nuclear subcompartment/substructure was observed. In some of the cells, these changes lasted even during insulin/IGF-1 replenishment (Figure 4).

Likewise, in non-transformed cells (astrocytes), replenishment of serum-free medium with insulin was sufficient to induce the re-appearance of PGAM in nucleoli. However, the process was faster than in malignant cells and we did not observe the cap-like structures; in contrast the mitotic figures were more numerous than in the malignant cells (Suppl. Figure 4A).

### The effect of drugs inhibiting ribosome biogenesis on PGAM subcellular localization

**Figure 5:**
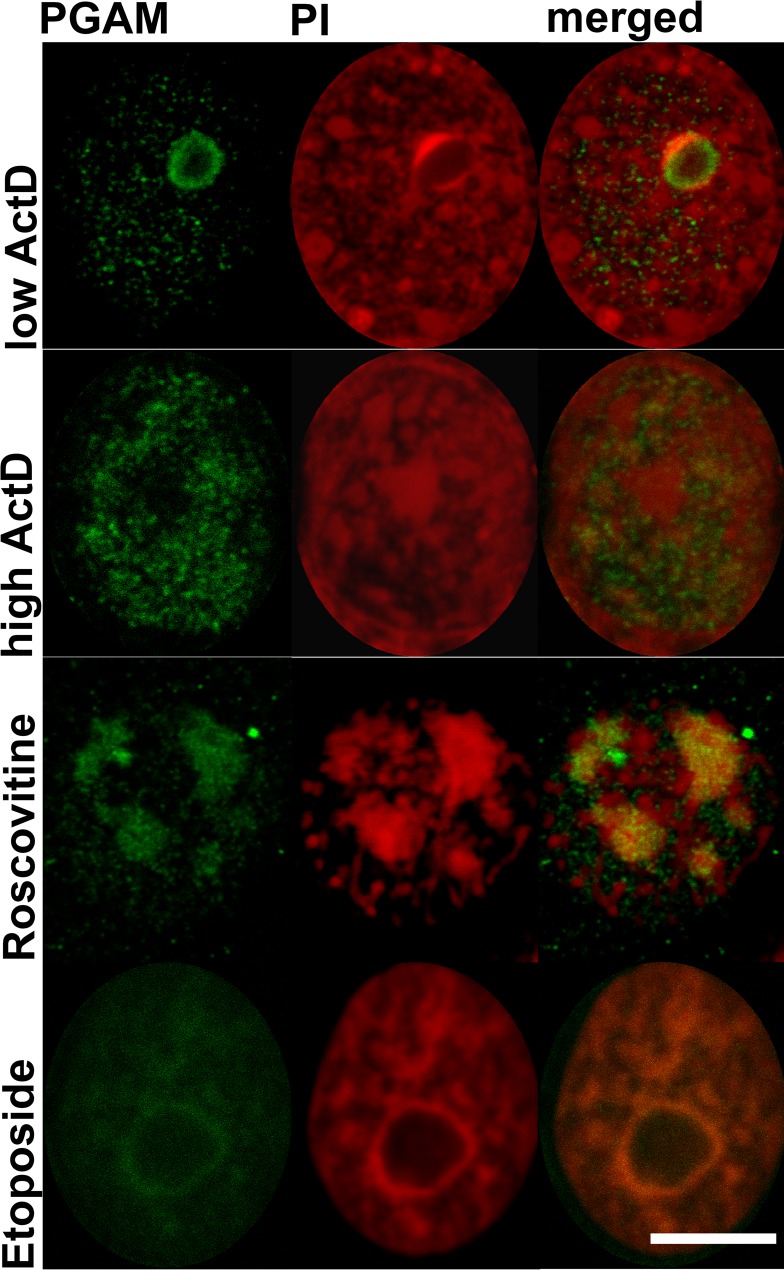
Localization of PGAM with antibody directed to the C-terminal peptide of the protein in nuclei of KLN-205 cells treated with drugs disturbing ribosomal biogenesis ActD – actinomycin D; PI – propidium iodide. Bar=5 μm.

Incubation of the KLN-205 cells with roscovitine which blocks proliferation acting as an inhibitor of cyclin-dependent kinases [7], resulted in spreading of nucleolar apparatus into necklace-like structures. In these conditions, while irregular in shape – but PGAM- and PI-positive, structures could still be observed (Figure 5).

After treatment of KLN-205 cells with etoposide – a topoisomerase II inhibitor inducing DNA damage but also influencing nucleolar protein content [8], a weak immunofluorescent signal of PGAM was observed in nuclei and in nucleolar cap-like structures (Figure 5). Central regions of nucleolus were devoid of PGAM staining.

After a brief exposure of the KLN-205 cells to low concentrations (0.04 μg/ml) of actinomycin-D (ActD) – which is known to selectively reduce rRNA synthesis and induce release of nucleolar protein to nucleoplasm [9], PGAM was redistributed to cap-like structures and was absent from central regions of nucleoli (Figure 5). These regions exhibited also lack of staining with PI, which suggests an absence of RNA there. Treatment of the cells with relatively high concentration of ActD (2.5 μg/ml) which completely blocks DNA transcription resulted in a rather homogeneous nuclear localization of PGAM, as determined with the use of antibodies against the C-terminus (Figure 5).

**Figure 6:**
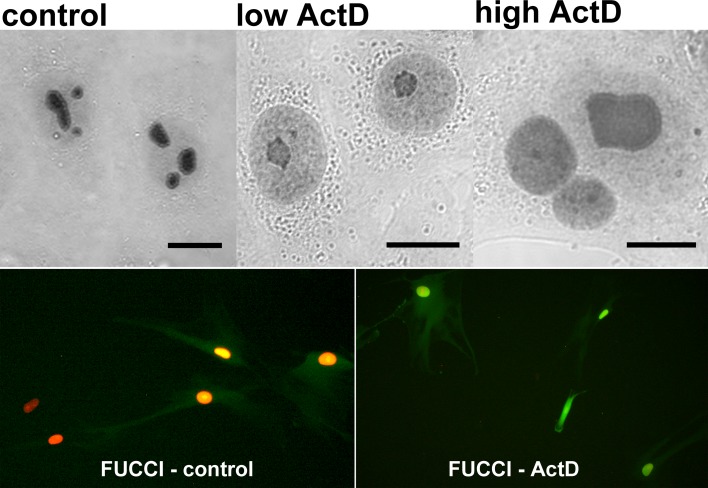
Correlation of ActD-stimulated changes of PGAM localization in NSCLC cells with blockade of the cell cycle cell in G2 phase Upper panel: the PGAM2 localization in the cells incubated with 0.04 μg/ml (low) or 2.5 μg/ml (high) actinomycin D (ActD). Lower panel: the cells in G1 (red) and G1/S (yellow) phase in control conditions and blockade of the ActD-treated cells in G2 phase (green fluorescence) as determined by FUCCI Cell Cycle Sensor. Bar=10 μm.

To determine if ActD action-associated export of PGAM from nucleoli correlated with changes in cell cycle phase we used the Premo™ FUCCI Cell Cycle Sensor which allows imaging of cell cycle progression. However, the KLN205 cells appeared to be hardly transfectable and we observed the sensor-related signal only in a small proportion of the cells. Thus, we decided to use the NSCLC cells, testing first response of their nucleolar pool of PGAM to ActD. As in the previous cells, such treatment of NSCLC resulted in redistribution of PGAM to nucleolar cap structures or in homogeneous nuclear presence of the enzyme, depending on ActD concentration (Figure 6).

NSCLC cells transfected with FUCCI reagent and exposed overnight to 0.04 μg/ml of ActD appeared to be blocked in the late G2 phase, since the nuclei and also cytoplasm of majority of the cells exibited intense green fluorescence. This was in line with the data published by Ma and Pederson [9] demonstrating that ActD elicits nucleolar stress and blocks cells in very late interphase. In the untreated NSCLC cells, the fluorescence of the majority of nuclei pointed to G1 (red fluorescence) or G1/S (yellow fluorescence) phase (Figure 6).

These observations indicated that a lack of nucleolar PGAM was correlated with the G2/M cell cycle blockade and inhibition of rRNA synthesis.

### Transfection of the KLN-205 cells with PGAM2-FITC

**Figure 7:**
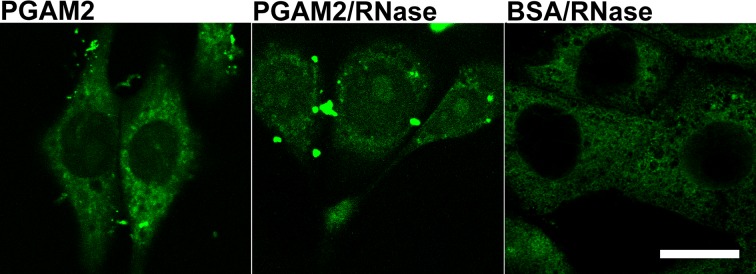
The effects of KLN-205 cells transfection with PGAM2 The cells were transfected with PGAM2-FITC in normal conditions and in the presence of RNase. As a control, cells were transfected with BSA-FITC. Bar=10 μm.

Transfection of the KLN-205 cells with fluorescently-labeled PGAM2 in the presence of serum in culture medium resulted in predominantly cytoplasmic accumulation of the enzyme (Figure 7). Labeling of nuclei was relatively weak and no specific localization in nuclear substructures was observed. Assuming that nucleolar PGAM2 is tightly bound in a protein and/or RNA complex, the lack of the native-to-labeled PGAM2 molecule exchange might point to high stability of this complex. When PGAM2-FITC was accompanied with RNase A, the PGAM2 molecules were more readily exchanged and nucleolar localization of the enzyme was observed (Figure 7). FITC-labeled BSA introduced into the KLN-205 cells (as a control) with the use of ProteoJuice, localized diffusely within cytoplasm of the cells (Figure 7).

### Silencing of PGAM2 gene expression

The antibodies against the C-terminus of PGAM do not allow the distinction between the brain (PGAM1) and muscle (PGAM2) isoform. PGAM2 isoform is much less abundant in tissues (except muscle tissue; Table 1) than PGAM1, and these low titers of PGAM2 may suggest that the isoform does not play any significant physiological role in a cell. However, concentration of PGAM2 is similar or higher than titers of proteins involved in ribosome biogenesis, e.g. DNA-directed RNA polymerase I (Suppl. Table 1).

**Table 1:** The concentration of PGAM isoforms in mouse tissues

Tissue	[PGAM1](pmol/mg)	[PGAM2](pmol/mg)	n	[PGAM1]/[PGAM2]
Brain	56 ± 14	0.86 ± 0.59	3	65
Ovary	32 ± 11	0.93 ± 0.23	3	35
Thymus	15 ± 7.8	0.21 ± 0.066	2	74
Thyroid	13 ± 2.9	34.9 ± 5.8	3	0.38
Red muscle	0.54 ± 0.14	97.23 ± 22.4	4	0.0055
White muscle	0.17 ±0.11	178 ± 26	4	0.00095

Thus, we used a commercially prepared siRNA to silence expression of the PGAM2 gene in KLN-205 cells and tested the effects of the silencing on structure and function of nucleoli.

PCR reaction with the use of PGAM1- and PGAM2-specific primers revealed about 3.7-fold decrease of the PGAM2 mRNA amount in the PGAM2-silenced cells as compared to control cells. No visible difference was observed in the amount of PGAM1 mRNA (Suppl. Figure 2).

**Figure 8:**
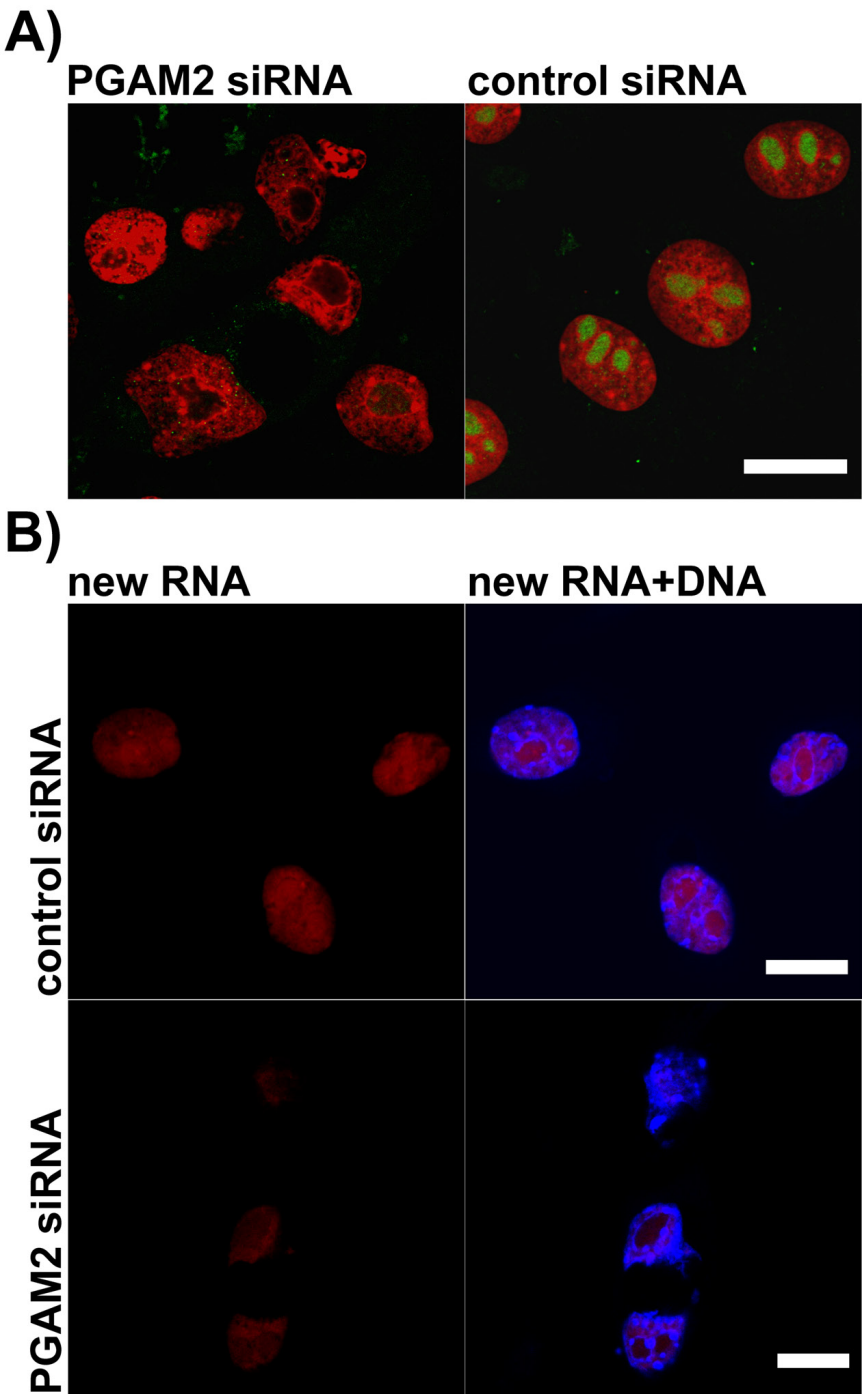
The effects of PGAM2 silencing on nucleolar morphology and new RNA production in KLN-205 cells **A.** in PGAM2-silenced cells, unlike control cells, stained with propidium iodide (red), only one centrally located nucleolus can be found and nucleolar PGAM staining (green) is undetectable; **B.** the level of new RNA (red) synthesis in PGAM2-silenced cells is reduced as compared to control cells. To visualize DNA the cells were counterstained with Hoechst 33342 (blue). Bar=10 μm.

Immunofluorescent reactions failed to detect PGAM in nucleoli of PGAM2-silenced cells (Figure 8A). This indicated that the PGAM2 subunit was indispensable for nucleolar localization of PGAM dimer and hence, nucleoli may contain both PGAM2 homodimers and PGAM2/PGAM1 heterodimers, but not PGAM1 homodimers.

In most of the cells with silenced PGAM2 expression, only one, centrally located nucleolus was observed (Figure 8A). Additionally, the shape of such nucleolus (and whole nucleus) was often distorted.

To determine if the decline of PGAM2 protein influenced nucleolar function, we examined the level of RNA production in PGAM2-silenced and control cells using a click chemistry–based fluorescence tagging procedure (see Methods). As a result, we observed a significant reduction of RNA signal resulting from nucleolar compartment of PGAM2- silenced cells (Figure 8B) pointing to diminished transcription of rDNA (i.e. diminished production of ribosomal RNA). The global level of RNA synthesis was also reduced. We also observed increased intensity of activated caspase-3 immunostaining (Suppl. Figure 3) and 4.6-fold decrease of mitotic index (not shown) in PGAM2- silenced cells compared to control cells.

Similar reduction of RNA synthesis was observed also in PGAM2-silenced non-malignant cells, HL-1 cardiomyocytes (Suppl. Figure 4B).

Moreover, 72 h after transfection with siRNA we observed almost 2-fold decrease of cell number and over 20% reduction of protein concentration per one cell in cultures of PGAM2-silenced cells (KLN-205 as well as HL-1), as compared to cultures of cells treated with control, non-silencing siRNA.

### PGAM-interacting nucleolar proteins

The MS analysis of PGAM-interacting proteins in nucleoli of KLN-205 cells revealed that the enzyme may interact with constituent proteins of 40S and 60S ribosomal complexes, proteins involved in ribosome biogenesis and post-translational modifications (e.g. with ribosome-associated chaperones, such as peptidyl-prolyl cis-trans isomerase B) and proteins linking the nucleolar stress with cell cycle regulation and apoptosis (e.g. RPS27a and L26 protein) [10-12] (Table 2). PGAM associates with several members of 14-3-3 adapter proteins family (14-3-3 zeta/delta, sigma, beta/alfa, teta) of which the yeast homolog – Bmh2 (http://www.yeastgenome.org/cgi-bin/protein#), was shown to attenuate the repression of genes involved in ribosome biogenesis [13].

**Table 2:** PGAM-interacting nucleolar proteins detected with affinity chromatography and subsequent mass spectrometry analysis

protein	score	sequence coverage (%)	accession number
14-3-3 zeta/delta sigma beta/alfa teta	213 188 183 105	10 11 12 8	P63101 O70456 Q9CQV8 P68254
40S ribosomal protein S16	188	11	P14131
60S acidic ribosomal protein P0	132	8	P14869
40 ribosomal protein S3	99	8	P62908
60S ribosomal protein L8	95	6	P62918
60S ribosomal protein L18	92	5	P35980
40S ribosomal protein S14	91	7	P62264
histone H3.3C	88	13	P02301
peptidyl-prolyl cis-trans isomerase B	71	6	P24369
peptidyl-prolyl cis-trans isomerase A	70	5	P17742
60S ribosomal protein L26	69	8	P61255
40S ribosomal protein S18	63	7	P62270
40S ribosomal protein S6	59	3	P62754
ubiquitin-40S ribosomal protein S27a	44	5	P62983
60S ribosomal protein L10a	43	7	P53026

## DISCUSSION

In the present paper, we have demonstrated, using several lines of evidence, that a glycolytic enzyme – phosphoglycerate mutase, is present in nucleoli of non-transformed as well as neoplastically transformed cells. The enzyme is directed to nucleoli of cancer cells by insulin/IGF-1–PI3K signaling pathway, and appears to interact with the ribosomal proteins. The lack of the nucleolar PGAM is correlated with G2/M cell cycle blockade and the silencing of PGAM2 gene expression resulted in structural changes within nucleoli, inhibition of RNA expression and the reduction of cellular protein content.

The nucleolus is the site of production of ribosomes. However, over the last years, proteomic studies have demonstrated the presence in the nucleolus of a large number of proteins which were not typically considered as a part of rDNA transcription and processing machinery. Most of these proteins regulate cell proliferation, stress response and aging [3].

Yet, among the nucleolar proteins, enzymes of carbohydrate metabolism – glycolysis and pentose phosphate pathway, have been found [4]. The physiological meaning of such localization has not been explained: both the “classical” metabolic role and the potential additional function of these enzymes in nucleolus remain unclear. It has been suggested that the nucleolus may act as a regulator of protein activity – just by their sequestration [14] However, since all glycolytic enzymes are highly abundant proteins in a cell [15], the “sequestration for inhibition” theory sounds improbable.

In the cytoplasm of cancer cells, the C-terminus of the PGAM molecules is involved in maintaining the enzyme in its active form [16] and in association with other enzymes of glycolytic complex and thus, it is unavailable to antibodies [2]. In the present work, we demonstrated that in nucleoli of not only cancer but also non-transformed cells, PGAM seems to be tightly surrounded by other proteins and/or nucleic acids and only the C-terminus of this enzyme is available to anti-PGAM antibodies (Figures 1, 2). The results of the protein transfection experiment suggest that this association is quite stable. Only the digestion of nucleolar structures with RNase made the whole PGAM protein more readily accesible to antibodies as well as to the process of exchange of the native to FITC-labeled PGAM molecules (Figure 1B and 7). This indicates that the rRNA presence is not prerequisite to the nucleolar localization of PGAM, although some interactions between the two components cannot be excluded. And since a tight interaction of C-terminal peptide of PGAM with its central part is needed to maintain the enzyme in the active form [16], thus the exposition of the C-terminus to antibodies suggests that nucleolar PGAM is constantly in its enzymatically inactive state. Previously, we have demonstrated that the nucleolar localization of PGAM was independent of concentration of glucose metabolites [2]. In the present work, we did not observe any of the glycolytic enzymes among the potential binding partners of nucleolar PGAM. Thus, together, the data suggests that the nucleolar role(s) of PGAM are different from the cytoplasmic functions and not related to the catalytic activity of this enzyme. However, to unequivocally prove this, further studies using catalytically inactive PGAM2 mutants are needed.

In the present paper, we showed that serum starvation, which results in cessation of proliferation, resulted also in withdrawal of PGAM from nucleoli (especially from their central regions), and that activation of the insulin/IGF-1–PI3K pathway in the serum-depleted cells was sufficient for nucleolar re-accumulation of PGAM (Figures 3, 4).

Serum starvation is known to cause disruption of rDNA transcription and ribosome biogenesis which finally results in the cell cycle arrest. It has been demonstrated that these changes are an effect of insulin deficiency. Insulin regulates the nuclear content of upstream binding factor (UBF) and RNA polymerase I-associated factor (PAF) 53 which are required for assembly of RNA polymerase I into an active complex. As a result, insulin stimulation rises the steady-state number of ribosomes within a cell [17]. It has been also shown that activity of the IGF-1–PI3K pathway is essential to induction of polymerase I [18].

Therefore, we tested the relationship between nucleolar PGAM and ribosome biogenesis. After silencing of PGAM2 expression in cells, complete lack of nucleolar PGAM immunoreactivity was observed (Figure 8A). This indicated that although both PGAM1 and PGAM2 isoforms were present in nucleoli (Suppl. Figure 1), the PGAM2 subunit was indispensable for nucleolar localization of the PGAM dimer. What was more intriguing, the absence of PGAM in nucleoli tightly correlated with reduction of the number of nucleoli (only one, large, centrally located, and often distorted nucleolus was observed in majority of the cells; Figures 4, 5, 8), inhibition of RNA synthesis (Figure 8, Suppl. Figure 4B), decrease of mitotic index and reduction of cellular protein content. This strongly suggests that the biogenesis of ribosomes depends on nucleolar localization of PGAM.

In search for further evidence that nucleolar localization of PGAM correlates with rDNA transcription and/or rRNA processing, we treated cells with chemotherapeutic drugs known to disturb ribosomal biogenesis at the level of transcription (ActD) and early or late rRNA processing (roscovitine and etoposide, respectively) [19]. A brief treatment of cells with low concentrations of ActD resulted in release of nucleolar proteins to nucleoplasm [20]. Under such conditions, nucleoli are depleted of RNA polymerase I, ribosomal proteins and exosome components [21] and the amount of nucleolar RNA is strongly diminished. We found that after the ActD treatment, PGAM immunoreactivity in central regions of nucleoli was significantly reduced and the enzyme was redistributed to nucleolar cap structures (Figure 5). This suggests that PGAM is a component of rDNA transcription machinery. On the other hand, inhibition of early rRNA processing with roscovitine resulted in spreading of nucleolar apparatus and localization of PGAM into so-called necklace-like structures (Figure 5). Although appearance of these structures is a sign of disruption of ribosome biogenesis, some synthesis of 45S rRNA must continue to maintain the necklace morphology [22]. Thus, the presence of PGAM2 in the necklace-like nucleoli might narrow the enzyme function to transcription of 45S rDNA.

The inhibition of late rRNA processing with etoposide resulted again in reduction in nucleolar PGAM immunoreactivity (Figure 5).

Such changes in PGAM nucleolar distribution during inhibition of pre-ribosome biogenesis indicates that the enzyme is involved in the proper functioning of all stages of pre-ribosome production machinery. In line with this, the mass spectrometry analysis of nucleolar PGAM binding partners revealed that PGAM may interact with several 40S and 60S ribosomal proteins (Table 2). Among potential interaction partners are ribosomal proteins with extra ribosomal functions, such as L26 and RPS27a. Both of these proteins in their free cytoplasmic form are involved, though by different mechanisms, in elevation of cellular levels of p53 protein and induction of apoptosis [10, 12]. Reduction of pre-ribosome integrity in the PGAM2-silenced cells might therefore increase the availability of these proteins and facilitate activation of the p53-dependent cell response to disturbance of ribosome biogenesis. The increased immunoreactivity of activated caspase-3 observed in PGAM2-silenced cells appears to support this hypothesis (Suppl. Figure 3). To the best of our knowledge, PGAM has not been found in mature ribosomes (http://www.lamondlab.com/NOPdb3.0) and hence, the role of the enzyme seem to be essential for biogenesis but not to further functioning of ribosomes.

Efficient ribosome biogenesis is crucial in animal cells undergoing rapid proliferation but it is also an energy-consuming process. Reduction of nutrient availability leads to decrease in ribosome subunit production. Thus, one can argue that silencing of PGAM2 can influence the nucleolar function just by reducing the glycolytic flux and cellular pool of ATP. However, as we demonstrated above (Table 1), in non-muscle cells, the PGAM2 isoform is much less abundant than PGAM1 protein and it is unlikely that this isoform may significantly affect the glycolytic flux. Therefore, the effect of PGAM2 silencing on the nucleolus is most probably not related to energy deprivation.

The canonical insulin and IGF-1 signaling pathway induced by PI3K is associated with regulation of cellular metabolism and survival. Targeting of this pathway is considered in the context of cancer treatment and there is accumulating evidence that mutation of *PI3KCA* (PI3K) contributes to the development of many types of malignancies [23-24]. Inhibition of the kinase suppresses metastasis, mainly through cytostatic effects. However, the effectiveness of treatment with a single PI3K inhibitor has been questioned [24]. Similarly, results of clinical trials using IGF-1 receptor-specific antibodies for cancer treatment are disappointing [25].

Both insulin and IGF-1 acting via PI3K (this work and [18], respectively) control ribosome biogenesis and thereby protein production and cell growth. According to our results, this effect is exerted partially through directing of PGAM2 to the nucleolus, which is a prerequisite for synthesis and initial assembly of new pre-ribosome subunits, before their transfer to the nucleus and cytoplasm. In the absence of nucleolar PGAM, the efficiency of processing and export of pre-ribosomal subunits is compromised, resulting in the distortion of the nucleolar structure and decline of cellular RNA and protein synthesis. Thus, direct targeting of PGAM2 transport to nucleoli might be worth considering as a part of a new anti-cancer combination therapy.

## MATERIALS AND METHODS

### Cell lines and chemicals

The murine squamous cell carcinoma line KLN-205 was obtained from ECACC, Europe. The HL-1 cardiomyocyte cell line was a gift from Dr. W.C. Claycomb (Louisiana State University Health Science Center, New Orleans, LA, USA). Goat antibodies specific to C-terminal peptide of PGAM (NB100-774) were from Novus Biologicals. ProteoJuice Protein Transfection Reagent was from Merck Millipore. Fluoshield Fluorescent Mounting Medium was from Dako. Lipofectamine^®^ 2000, Premo™ FUCCI Cell Cycle Sensor and Click-iT^®^ RNA Alexa Fluor^®^ 594 Imaging Kit were from Life Technologies. NHS-SS-Diazirine cross-linker (spacer arm length 1.35 nm) was from Thermo Scientific Pierce; protein G-agarose from Roche; siRNA-A (sc-37007) and mouse PGAM2 siRNA (sc-152183) were obtained from Santa Cruz Biotechnology. Cell culture media and supplements, anti-rabbit FITC-conjugated (F6005) and anti-goat FITC-conjugated (F9012) antibodies and all other reagents were purchased from Sigma.

### Cell culture

The KLN-205 cells were cultured on coverslips in Eagle's Minimal Essential Medium supplemented with L-glucose (5 mM), L-glutamine (2 mM), 10% (v/v) non-essential amino acids, penicillin (100 units/ml), streptomycin (100 mg/ml), and 10% (v/v) fetal bovine serum (standard medium) at 37°C and in 5% CO2 atmosphere. To test the effect of various biological signals on nuclear localization of PGAM, after 48 h of the KLN-205 cells culture, the standard culture medium was replaced with serum-free medium for 48 h. Then the cells were cultured in the serum-free medium supplemented with insulin (0.2 μM), insulin and wortmannin (the inhibitor of phosphoinositide 3-kinase; 1μM) or with IGF-1 (0.013 μM) for 24 h and localization of PGAM was examined using immunofluorescent methods (as described below). Alternatively, the KLN-205 cells were cultured for 2 hours in the standard medium supplemented with actinomycin D (0.04 μg/ml or 2.5 μg/ml), roscovitine (20 μM) or etoposide (300 μM).

Human non-small cell lung carcinoma (NSCLC) cells were cultured as described in [26]. All the procedures used to acquire the cells were approved by the Commission of Bioethics at Wroclaw Medical University. HL-1 cardiomyocyte cell line was cultured as described before [27]. Mouse astrocytes were isolated and cultured as described in [28]. The protocol of isolation of mouse cells and tissues was approved by the II Local Scientific Research Ethical Committee, Wroclaw University of Environmental and Life Sciences (permission #118/2010). These cells were used in the experiments of PGAM localization by immunofluorescent or immunoperoxidase method.

### Tissue sections

Histologically proven archival human breast cancer tissue samples embedded in paraffin were kindly provided by Piotr Dziegiel from Department of Histology and Embryology, Wroclaw Medical University, Wroclaw, Poland. PGAM localization in the slices was examined by immunoperoxidase method using goat antibodies specific to C-terminal peptide of PGAM, anti-goat antibodies conjugated to biotin and ExtrAvidin®−Peroxidase. The 3,3-diaminobenzidine was used as a peroxidase substrate.

### RNase treatment

The cells cultured in the presence or in the absence of serum were briefly treated with 0.01% (v/v) Triton X-100 in Hanks Balanced Salt Solution (HBSS), washed thoroughly with HBSS and incubated with 1mg/ml RNase A for 30 minutes at RT. Then the cells were washed, fixed in paraformaldehyde and localization of PGAM was determined using the immunofluorescent method.

### Immunofluorescence and confocal microscopy

The cells were fixed in 4% paraformaldehyde and permeabilized in 0.1% (v/v) Triton X-100 in PBS. After incubation with 5% (w/v) BSA in PBS which blocks unspecific interactions of antibodies with cells, the cells were incubated with goat antibodies specific to C-terminal peptide of PGAM (0.5 μg/ml) or with rabbit anti-PGAM serum (100 μg/ml) directed against the whole PGAM molecule (produced and tested as described in [29] and with FITC-conjugated secondary antibodies (1:1000). Then the cells were counterstained with propidium iodide (PI, 0.2 μg/ml), embedded in Fluoshield mounting medium and examined with the FV1000 confocal microscope (Olympus) equipped with diode lasers, the Plan Apo 60×/1.4NA Oil objective and appropriate filter sets. All images were acquired using the Sequential Scan option. In control reactions, the primary antibodies were omitted.

### PGAM2 purification and fluorescent labeling

Phosphoglycerate mutase from rabbit skeletal muscle was purified to homogeneity according to [29]. Fluorescently labeled enzyme (PGAM2-FITC) was obtained by modification of the protein with fluorescein isothiocyanate (FITC) as described by Goding [30]. The lack of proteolysis of the labeled protein was determined by SDS–PAGE. The number of fluorochrome molecules conjugated to the enzyme was estimated spectrophotometrically to be 1.9 FITC molecules per monomer of PGAM2.

### Transfection of cells with PGAM2-FITC

Transfection of the KLN-205 cells with 3 μg of FITC-labeled rabbit muscle PGAM2 (the amino-acid sequences of rabbit and mouse PGAM2 show 95% identity) was performed using the ProteoJuice Protein Transfection Reagent as described previously [31]. After 3 hours of cell transfection performed in serum-free culture medium, the medium was supplemented with 5% serum for 12 hours. Then the cells were washed, fixed in paraformaldehyde, counterstained with propidium iodide and examined using the confocal microscope. Alternatively, to facilitate the exchange of PGAM2-FITC with native PGAM molecules within nucleolar structures, the cells were transfected with 3 μg of PGAM2-FITC and 1μg of RNase A. In control experiments, FITC-conjugated BSA was used instead of PGAM2-FITC.

### Isolation of nucleoli and identification of PGAM by mass spectrometry

Nucleoli were isolated from the KLN-205 cells cultured in the presence or absence of serum according to method described in [32], and analyzed using nano-high performance liquid chromatography combined with tandem mass spectrometry (nano-HPLC/MS/MS) with Amazon ETD mass spectrometer (Bruker Daltonik, Bremen, Germany) as described earlier [33]; for detailed methods see in Supplementary data 1).

### Identification of PGAM-interacting proteins

To identify PGAM-interacting protein in nucleoli, KLN-205 cells grown on a 100 mm Petri dishes were treated with a membrane-permeable cross-linker as we described previously [34]. Subsequently, nucleoli were isolated and nucleolar proteins were extracted according to [35]. The obtained nucleolar extract was incubated with the antibodies against C-terminal region of PGAM bound to protein G-agarose beads, overnight at 4°C, under agitation. Subsequently, the beads were thorougly washed to remove any unspecifically bound proteins and the PGAM–cross-linked proteins complexes were eluted with the urea buffer (20 mM Tris pH 7.5, RT, 100 mM NaCl, 8 M urea). The complexes were then analysed comercially with the ESI–MS at the Mass Spectrometry Laboratory (Institute of Biochemistry and Biophysics, Polish Academy of Sciences, Warsaw).

### Silencing of PGAM2 gene expression

The commercially prepared mouse PGAM2 siRNA was used to silence the PGAM2 gene expression in KLN205 and HL-1 cells, according to the manufacturer's instructions. As a control, the siRNA-A consisting of a scrambled sequence that does not lead to the specific degradation of any known cellular mRNA was used. Expression of PGAM1 and PGAM2 genes in these cells was monitored by PCR. For details see Supplementary data 2.

### RNA synthesis

Detection of global RNA synthesis in KLN-205 cells (untreated and with the silenced expression of PGAM2 gene) was performed using the Click-iT^®^ RNA Alexa Fluor^®^ 594 Imaging Kit, according to procedure provided by the manufacturer, and visualized with the confocal microscope. The cells were fed with 5-ethynyl uridine which was actively incorporated in the newly synthesized RNA. The level of this synthesis was then detected with azide-modified fluorochrome, Alexa Fluor^®^ 594. DNA was counterstained with Hoechst 33342 provided with the kit.

### Imaging of cell cycle stage

NSCLC cells were grown on coverslips to 70% confluence and then transduced with Premo™ FUCCI Cell Cycle Sensors (80 particles per cell) according to manufacturer's instruction. For induction of nucleolar stress, cells were exposed to 0.04 μg/ml ActD as described above. Then they were fixed with paraformaldehyde and examined with confocal microscope.

### The titers of PGAM isozymes in mouse tissues

Concentrations of PGAM1 and PGAM2 in whole cell tissue lysates were calculated by means of the ‘Total Protein Approach’ [36] using recently published datasets [37-38].

## SUPPLEMENTARY MATERIAL FIGURES AND TABLE



## References

[1] Zhang J, Yu L, Fu Q, Gao J, Xie Y, Chen J, Zhang P, Liu Q, Zhao S (2001). Mouse phosphoglycerate mutase M and B isozymes: cDNA cloning, enzyme activity assay and mapping. Gene.

[2] Kowalski W, Nocon D, Gamian A, Kolodziej J, Rakus D (2012). Association of C-terminal region of phosphoglycerate mutase with glycolytic complex regulates energy production in cancer cells. J Cell Physiol.

[3] Boisvert FM, van Koningsbruggen S, Navascués J, Lamond AI (2007). The multifunctional nucleolus. Nat Rev Mol Cell Biol.

[4] Jarboui MA, Bidoia C, Woods E, Roe B, Wynne K, Elia G, Hall WW, Gautier VW (2012). Nucleolar protein trafficking in response to HIV-1 Tat: rewiring the nucleolus. PLoS One.

[5] Takeda K, Komuro Y, Hayakawa T, Oguchi H, Ishida Y, Murakami S, Noguchi T, Kinoshita H, Sekine Y, Iemura S, Natsume T, Ichijo H (2009). Mitochondrial phosphoglycerate mutase 5 uses alternate catalytic activity as a protein serine/threonine phosphatase to activate ASK1. Proc Natl Acad Sci U S A.

[6] Banski P, Kodiha M, Stochaj U (2011). Exploring the Nucleolar Proteome: Novel Concepts for Chaperone Trafficking and Function. Current Proteomics.

[7] Meijer L, Borgne A, Mulner O, Chong JP, Blow JJ, Inagaki N, Inagaki M, Delcros JG, Moulinoux JP (1997). Biochemical and cellular effects of roscovitine, a potent and selective inhibitor of the cyclin-dependent kinases cdc2, cdk2 and cdk5. Eur J Biochem.

[8] Boisvert FM, Lamond AI (2010). p53-Dependent subcellular proteome localization following DNA damage. Proteomics.

[9] Ma H, Pederson T (2013). The nucleolus stress response is coupled to an ATR-Chk1-mediated G2 arrest. Mol Biol Cell.

[10] Nosrati N, Kapoor NR, Kumar V (2015). DNA damage stress induces the expression of Ribosomal Protein S27a gene in a p53-dependent manner. Gene.

[11] Wang H, Yu J, Zhang L, Xiong Y, Chen S, Xing H, Tian Z, Tang K, Wei H, Rao Q, Wang M, Wang J (2014). RPS27a promotes proliferation, regulates cell cycle progression and inhibits apoptosis of leukemia cells. Biochem Biophys Res Commun.

[12] Ofir-Rosenfeld Y, Boggs K, Michael D, Kastan MB, Oren M (2008). Mdm2 regulates p53 mRNA translation through inhibitory interactions with ribosomal protein L26. Mol Cell.

[13] Trembley MA, Berrus HL, Whicher JR, Humphrey-Dixon EL (2014). The yeast 14-3-3 proteins Bmh1 and Bmh2 differentially regulate rapamycin-mediated transcription. Biosci Rep.

[14] Visintin R, Amon A (2000). The nucleolus: the magician's hat for cell cycle tricks. Curr Opin Cell Biol.

[15] Wisniewski JR, Koepsell H, Gizak A, Rakus D (2015). Absolute protein quantification allows differentiation of cell specific metabolic routes and functions. Proteomics.

[16] Walter RA, Nairn J, Duncan D, Price NC, Kelly SM, Rigden DJ, Fothergill-Gilmore LA (1999). The role of the C-terminal region in phosphoglycerate mutase. Biochem J.

[17] Hannan KM, Rothblum LI, Jefferson LS (1998). Regulation of ribosomal DNA transcription by insulin. Am J Physiol.

[18] James MJ, Zomerdijk JC (2004). Phosphatidylinositol 3-kinase and mTOR signaling pathways regulate RNA polymerase I transcription in response to IGF-1 and nutrients. J Biol Chem.

[19] Burger K, Mühl B, Harasim T, Rohrmoser M, Malamoussi A, Orban M, Kellner M, Gruber-Eber A, Kremmer E, Hölzel M, Eick D (2010). Chemotherapeutic drugs inhibit ribosome biogenesis at various levels. J Biol Chem.

[20] Rubbi CP, Milner J (2003). Disruption of the nucleolus mediates stabilization of p53 in response to DNA damage and other stresses. EMBO J.

[21] Andersen JS, Lam YW, Leung AK, Ong SE, Lyon CE, Lamond AI, Mann M (2005). Nucleolar proteome dynamics. Nature.

[22] Granick D (1975). Nucleolar necklaces in chick embryo fibroblast cells. II. Microscope observations of the effect of adenosine analogues on nucleolar necklace formation. J Cell Biol.

[23] Davis NM, Sokolosky M, Stadelman K, Abrams SL, Libra M, Candido S, Nicoletti F, Polesel J, Maestro R, D'Assoro A, Drobot L, Rakus D, Gizak A, Laidler P, Dulińska-Litewka J, Basecke J, Mijatovic S, Maksimovic-Ivanic D, Montalto G, Cervello M, Fitzgerald TL, Demidenko Z, Martelli AM, Cocco L, Steelman LS, McCubrey JA (2014). Deregulation of the EGFR/PI3K/PTEN/Akt/mTORC1 pathway in breast cancer: possibilities for therapeutic intervention. Oncotarget.

[24] Garrett JT, Chakrabarty A, Arteaga CL (2011). Will PI3K pathway inhibitors be effective as single agents in patients with cancer?. Oncotarget.

[25] Pollak M (2012). The insulin and insulin-like growth factor receptor family in neoplasia: an update. Nat Rev Cancer.

[26] Sok AJ, Gizak A, Mamczur P, Piotrowska A, Knapik A, Kolodziej J, Dziegiel P, Wisniewski JR, Rakus D (2014). Demethylation with 5-Aza-2-deoxycytidine Affects Oxidative Metabolism in Human and Mouse Non-small Cell Lung Cancer Cells. J Cancer Sci Ther.

[27] Gizak A, Zarzycki M, Rakus D (2009). Nuclear targeting of FBPase in HL-1 cells is controlled by beta-1 adrenergic receptor-activated Gs protein signaling cascade. Biochim Biophys Acta.

[28] Mamczur P, Borsuk B, Paszko J, Sas Z, Mozrzymas J, Wisniewski JR, Gizak A, Rakus D (2015). Astrocyte-neuron crosstalk regulates the expression and subcellular localization of carbohydrate metabolism enzymes. Glia.

[29] Kowalski W, Gizak A, Rakus D (2009). Phosphoglycerate mutase in mammalian striated muscles: subcellular localization and binding partners. FEBS Lett.

[30] Goding JW (1976). Conjugation of antibodies with fluorochromes: modifications to the standard methods. J Immunol Methods.

[31] Gizak A, Maciaszczyk-Dziubinska E, Jurowicz M, Rakus D (2009). Muscle FBPase is targeted to nucleus by its 203KKKGK207 sequence. Proteins.

[32] Andersen JS, Lyon CE, Fox AH, Leung AK, Lam YW, Steen H, Mann M, Lamond AI (2002). Directed proteomic analysis of the human nucleolus. Curr Biol.

[33] Waluk DP, Sucharski F, Sipos L, Silberring J, Hunt MC (2012). Reversible lysine acetylation regulates activity of human glycine N-acyltransferase-like 2 (hGLYATL2): implications for production of glycine-conjugated signaling molecules. J Biol Chem.

[34] Gizak A, Pirog M, Rakus D (2012). Muscle FBPase binds to cardiomyocyte mitochondria under glycogen synthase kinase-3 inhibition or elevation of cellular Ca2+ level. FEBS Lett.

[35] Chamousset D, Mamane S, Boisvert FM, Trinkle-Mulcahy L (2010). Efficient extraction of nucleolar proteins for interactome analyses. Proteomics.

[36] Wisniewski JR, Ostasiewicz P, Dus K, Zielinska DF, Gnad F, Mann M (2012). Extensive quantitative remodeling of the proteome between normal colon tissue and adenocarcinoma. Mol Syst Biol.

[37] Wisniewski JR, Hein MY, Cox J, Mann M (2014). A “proteomic ruler” for protein copy number and concentration estimation without spike-in standards. Mol Cell Proteomics.

[38] Rakus D, Gizak A, Deshmukh A, Wisniewski JR (2015). Absolute Quantitative Profiling of the Key Metabolic Pathways in Slow and Fast Skeletal Muscle. J Proteome Res.

