# Supplementary material for: Insulin/IGF1-PI3K-dependent nucleolar localization of a glycolytic enzyme – phosphoglycerate mutase 2, is necessary for proper structure of nucleolus and RNA synthesis
Source: Oncotarget. 2015 May 8;6(19):17237–50. doi: 10.18632/oncotarget.4044 (PMC4627304; doi:10.18632/oncotarget.4044)
Supplement: Supplementary file 1 [file oncotarget-06-17237-s001.pdf]

Insulin/IGF1-PI3K-dependent nucleolar localization of a glycolytic enzyme – phosphoglycerate mutase 2, is necessary for proper structure of nucleolus and RNA synthesis

Supplementary Material

Supplementary data 1.

Identification of PGAM in isolated nucleoli of KLN-205 cells using Nano high performance liquid chromatography combined with tandem mass spectrometry (nano-HPLC/MS/MS) and database analysis

Nucleoli were isolated from the KLN-205 cells cultured in the presence or in the absence of serum. Then the nucleolar samples were supplemented with protease and phosphatase inhibitors. Proteins were extracted with ice cold acetone and re-dissolved in 20 mM Tris-HCl, pH 8.3; 2.5 mM EDTA. TCEP (tris(2-carboxyethyl)phosphine) (Thermo Scientific) was added to a final concentration of 10 mM for 1 hour at 37°C to reduce disulfide bonds. Samples were treated with iodoacetamide (final concentration 30 mM) for 1 h at ambient temperature. Trypsin (Pierce Biotechnology, Rockford, IL, USA) was added to each sample (1:30 v/v) and digestion was performed overnight at 37°C. Samples were desalted on Harvard microspin C18 Vydac columns (Harvard Apparatus) and dried in a Speedvac concentrator.

All data were acquired on an Amazon ETD mass spectrometer (Bruker Daltonik, Bremen, Germany). Chromatographic separation of peptides was achieved on a Proxeon EASY-nLCsystem, equipped with 10 cm long, 3µm ReproSil-Pur C18 resin, 100 µm fused silica column, and 2 cm long, 5µm ReproSil-Pur C18 resin, 100 µm ID precolumn. The LC system operated with mobile phases: solvent A (98:2 H_2O:Acetonitrile (ACN)) (v:v), and solvent B (20:80 H_2O:ACN) (v:v) both supplemented with 0.1% formic acid. Samples were loaded from a cooled (7°C) auto sampler and separated with a linear gradient that was formed at a flow rate of 300 nL/min. It consisted of 100 min linear ramp up to 50% solvent B, followed by 75% B for 10 min, 10 min isocratic run at 75% B, and a 10 min isocratic run at 100% solvent A for system equilibration. The scan range was set to 300 – 1500 *m/z* in the MS mode, and was increased up to 3000 *m/z* in MS^2 mode. The instrument was operated in a positive ion-, and auto-MS^n ETD-NL modes. The three highest peaks within the range of 300-1500 *m/z* were automatically fragmented when their intensities exceeded a threshold value. Each precursor ion was fragmented with CID (collision induced dissociation). When neutral loss of 32.67 or 49 *Da* was detected at MS^2 level, the additional CID fragmentation was automatically triggered without previous isolation (*pseudo*-MS^3 was performed).

Additionally, all precursor ions with the observed neutral loss were subjected to ETD fragmentation, working with maximum acquisition time of 10 ms and reaction time of 100 ms. The maximum ETD precursor was set to 1200 m/z. LC-MS/MS spectra were analyzed using Proteinscape Software (Bruker Daltonik, Bremen, Germany). Peak lists were submitted to the Mascot Server 2.3 (Matrix Science, London, UK). Searches were performed using a database containing eleven sequences of PGAM isoforms [1]. Trypsin was selected as a site-specific enzyme for digestion. The precursor ion tolerance was set to 0.5 Da (#13C=1) and fragment mass tolerance was set to 1.3 Da. In addition, cysteine was defined with fixed carboxyamidomethylation modification (+57.0214 Da), serine/threonine as well as tyrosine were set with phosphorylation as variable modification (+79.9663 Da). Finally, y and b fragment ions were defined for all CID data, while c and z fragment ions were defined for ETD. Annotation of MS^2 (MS/MS) spectra was done using Proteinscape, and mass chromatograms were manually inspected with Data Analysis 4.0 (Bruker Daltonik, Bremen, Germany).

Supplementary Fig. 1.

PGAM-isoform-specific peptide sequences obtained from mass spectrometry analysis of isolated nucleoli of KLN-205 cells

Peptides detected in nucleoli of the cells cultured in full medium are marked in blue and bolded; peptides identified in nucleoli of the cells cultured in serum-free conditions are highlighted in yellow.

PGAM 5 (Q8BX10):

MAFRQALQLAACGLAGGSAAVLFSAVAVGKPRGGGDADTR**ATEPPAWTGAR**AGRGVWDTNWDRREPLSLINLKKR
NVESGEDELTSRLDHYKAKATRHIFLIRHSQYHVDGSLEKDR**TLTPLGREQAELTGLRLASLGLK**FNK**IVHSSMT**
RAVETTDIISKHLPGVSR**VSTDLLREGAPIEPDPPVSHWKPEAVQYYEDGA**RIEAAFRNYIHRADARQEEDSYEI
FICHANVIRYIVCRALQFPPEGWLRLSLNNGSITHLVIRPNGRVALRTLGDTGFMPPDKITRS

PGAM 1 (Q9DBJ1):

MAAYKLVLIRHGESAWNLENRFSGWYDADLSPAGHEEAKRGGQALRDAGYEFDICFTSVQKRAIRTLWTVLDAID
QMWLPVVRTWRLNERHYGGLTGLNK**AETAAKHGEAQVK**IWRRSYDVPPPPMEPDHPFYSNISKDR**RYADLTEDQL**
PSCESLKDTIARALPFWNEEIVPQIKEGKRVLIAAHGNSLRGIVKHLEGLSEEAIMELNLPTGIPIVYELDKNLK
PIKPMQFLGDEETVRKAMEAVAAQGKVKK

PGAM 2 (O70250):

MTTHRLVMVR**HGESLWNQENRFCGWFDAELSEKGAEEAK**R**GATAIK**DAKIEFDICYTSVLKRAIRTLWTILDVTD
QMWVPVVRTWRLNERHYGGLTGLNKAETAAKHGEEQVKIWRRSFDTPPPPMDEKHNYYTSISKDRRYAGLKPEEL
PTCESLKDTIARALPFWNEEIAPKIKAGQRVLIAAHGNSLRGIVKHLEGMSDQAIMELNLPTGIPIVYELDQNLK
PTKPMRFLGDEETVRKAMEAVAAQGKAK

Supplementary data 2.

Monitoring of PGAM2 silencing

The KLN-205 cells growing in 12-well culture dish were transfected with 0.75 µg of the siRNA per well using Lipofectamine and tested 72 h after transfection. Total RNA isolated from the KLN205 cells was transcribed with High-Capacity cDNA Reverse Transcription Kit (Genomed) and amplified with the PCR using primers (Genomed) specific for mouse PGAM1 (forward 5'-TTGCGAGATGCTGGCTATGA, reverse 5'-CCCATCTGCAGCTACAACTT) and PGAM2 (forward 5'-AAGCCTGAGGAGCTGCCTA, reverse 5-CTGTGCGGAAGTAACTTTATT). To normalize data, primers specific for mouse hypoxanthine phosphoribosyltransferase 1 (HPRT1; forward 5'-GCTTTCCCTGGTTAAGCAGTA, reverse 5'-CCTGTATCCAACACTTCGAGA) were used. PCR products were analyzed using agarose gel electrophoresis with ethidium bromide staining. Band intensities were quantified using GeneSnap and GeneTools software (Syngene).

Supplementary Fig. 2.

Verification of PGAM2 silencing by PCR

PCR was performed with primers specific for PGAM1, PGAM2 and HPRT1 (reference gene) using cDNA transcribed from total cellular RNA of the KLN-205 cells treated with siRNA specific for mouse PGAM2 and cells treated with control siRNA. For each reaction 5 and 10 µl of the product was loaded on separate lanes.

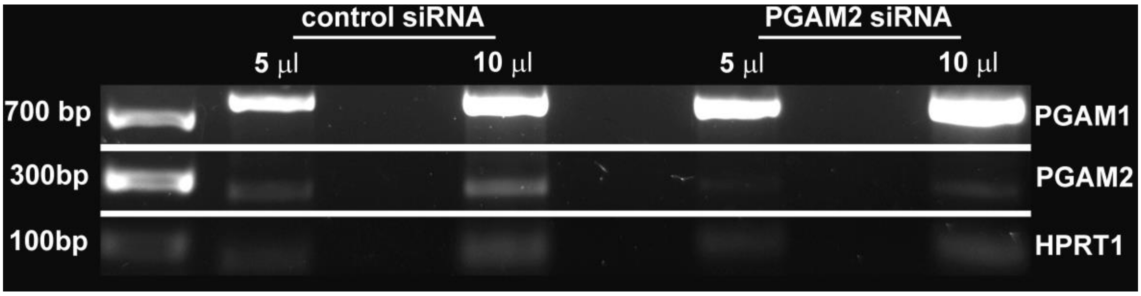

Supplementary data 3.

The effect of PGAM2 silencing on the activated caspase-3 immunoreactivity of KLN-205 cells

The PGAM2- and control-siRNA-treated KLN-205 cells were fixed in 4% paraformaldehyde, blocked with 5% (w/v) BSA in PBS and incubated with rabbit anti-active Caspase-3 antibody (ab2302, Abcam; 1 µg/ml) and then with Alexa Fluor® 633 anti-rabbit antibody (A21070, Life Technologies; 10 µg/ml). The cells were embedded in Fluoshield mounting medium and examined with the FV1000 confocal microscope (Olympus).

Supplementary Fig. 3.

Immunodetection of the activated caspase-3 in KLN-205 cells

(A) PGAM2-silenced cells; (B) control cells. Bar=10 µm.

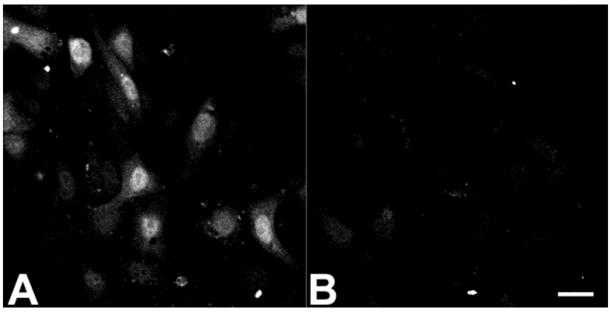

Supplementary Table 1.

The comparison of tissue concentrations of PGAM2 and DNA-directed RNA polymerase I subunits (Pol1a, Pol1b and Pol1c) which are crucial for ribosome biogenesis.

protein	brain		liver	
	concentration pmol/mg protein	std	concentration pmol/mg protein	std
PGAM2	0.885	0.488	0.101	0.047
Pol1a	0.023	0.002	0.022	0.013
Pol1b	0.059	0.004	0.093	0.006
Polr1c	0.057	0.002	0.053	0.017

Supplementary Fig. 4.

Regulation of nucleolar accumulation of PGAM and effect of PGAM2 expression silencing in non-transformed cells.

(A) Insulin induces re-accumulation of PGAM in nucleoli of serum-starved astrocytes. Bar=10 µm.

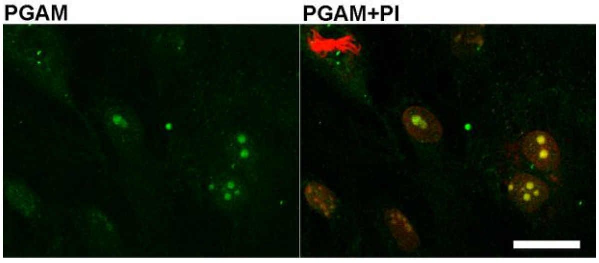

(B) PGAM2 silencing reduces new RNA production in HL-1 cardiomyocytes. Bar=5 µm.

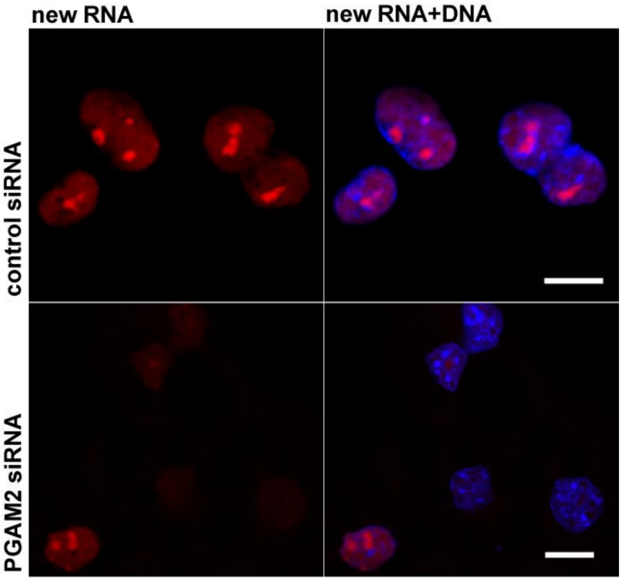

REFERENCES

1. Waluk DP, Sucharski F, Sipos L, Silberring J, Hunt MC. Reversible lysine acetylation regulates activity of human glycine N-acyltransferase-like 2 (hGLYATL2): implications for production of glycine-conjugated signaling molecules. J Biol Chem. 2012; 287: 16158-67.
